# The Effects of Guizhi Fuling Capsule Drug Serum on Uterine Leiomyoma Cells and Its Mechanism

**DOI:** 10.1155/2016/2393640

**Published:** 2016-11-08

**Authors:** Qi Shen, Weijing Ye, Xiaoli Hu, Chuchu Zhao, Lulu Zhou, Xueqiong Zhu

**Affiliations:** Department of Obstetrics and Gynecology, The Second Affiliated Hospital of Wenzhou Medical University, Wenzhou 325027, China

## Abstract

*Aims.* To observe the effects of Guizhi Fuling Capsule (GZFLC) drug serum on uterine leiomyoma cells and explore its mechanism.* Main Methods*. Sixty Sprague Dawley rats were randomly divided into two groups (normal saline lavage group and GZFLC lavage group), then, respectively, blank serum and GZFLC drug serum were collected, and finally human uterine leiomyoma cells were treated. Human leiomyoma tissues were collected from 20 patients who underwent uterine leiomyomas operations, and leiomyoma cells were primary cultured. The leiomyoma cells were treated by GZFLC drug serum in different concentrations (10%, 20%, and 30%) and variable treatment time (12 h, 24 h, 36 h, 48 h, and 72 h). Cell proliferation was observed using CCK8 assay. Flow cytometry and Annexin V/PI were used to assay the effects of GZFLC drug serum on cell apoptosis. Western blot analysis was used to assay the effects of GZFLC drug serum on TSC2, FOXO, and 14-3-3*γ* expression in uterine leiomyoma cells.* Key Findings*. In the concentrations of 10%*～*30%, GZFLC drug serum could inhibit proliferation of leiomyoma cells in dose-dependent manner; at the time of 36 h, cell inhibition rate was at the peak; GZFLC drug serum could induce apoptosis of leiomyoma also in a dose-dependent manner, and apoptosis rate quickly achieved maximum at 12 h time points, and then second apoptosis peak appeared at 36 h. Compared to nontreatment group, TSC2, FOXO, and 14-3-3*γ* expressions in drug serum group were significantly changed after 12 h treatment.* Significance*. GZFLC drug serum can efficiently inhibit the proliferation and induce apoptosis of leiomyoma cells, which is related to the 14-3-3*γ* pathway.

## 1. Introduction

Uterine leiomyomas are the most frequent neoplasm, affecting 20–40% of reproductive-age women [1]. Although frequently asymptomatic, leiomyomas can cause menorrhagia, abnormal uterine bleeding, pelvic pain or pressure, infertility, and miscarriage. Current treatments include surgical approaches and medical therapies (such as gonadotropin-releasing hormone agonists and hormonal therapies) [2]. However, surgery has some risks and is not suitable for patients who refuse operation [3]. Medical therapy also has some limitations. Gonadotropin-releasing hormone agonists can relieve leiomyoma-related symptoms but might have significant menopausal side effects [4, 5]. Progesterone antagonists, such as mifepristone, significantly reduce uterine and leiomyoma volume and alleviate leiomyoma-related symptoms without major adverse events. However, progesterone antagonists and other hormonal therapies which change progesterone and estrogen production could affect women's fertility function [6]. Therefore, safer and more effective therapy is needed for uterine leiomyomas.

Guizhi Fuling Capsule (GZFLC), as a famous traditional Chinese herbal formula, consisted of five herbs:* Cortex Moutan*,* Radix Paeoniae*,* Ramulus Cinnamomi*,* Poria cocos*, and* Semen Persicae* [7]. About 1800 years ago, it was first presented in* Jin Gui Yao Lue* (*Essential Prescriptions from the Golden Cabinet*) by the Chinese doctor Zhang Zhongjing. It has been widely used for the treatment of different diseases such as gynecological diseases and atherosclerosis [8, 9] and is involved in multiple pharmacological activities, such as stimulating the proliferative lesion soft and absorption, enhancing immune response, and preventing cancer cell growth [10]. In recent years, a clinical research confirmed that GZFLC was effective in uterine leiomyomas [11, 12]. GZFLC, the clinical recommended regimen, is taken orally as 3 capsules (in total 0.93 g) at one time, 3 times per day, 3 to 6 months, used alone or combined with mifepristone, GnRH-a, or other drugs to treat uterine leiomyomas. However, the treatment mechanism is not clear so far.

14-3-3 proteins, as a highly conserved phosphoserine/threonine-binding proteins family, comprised 7 isoforms (*β*, *γ*, *ε*, *η*, *σ*, *τ*/*θ*, and *ξ*) and participate in many physiological processes including cycle progression, transcriptional regulation, cell apoptosis, and proliferation [13–15]. In our previous study, via proteomics, we found that the expression of 14-3-3*γ* was reduced in uterine leiomyoma compared with normal myometrium [16]. Besides, 14-3-3*γ* is the receptor of FOXO and TSC2, playing an important role in cell proliferation and apoptosis via preventing them from dephosphorylation to control the proportion of cytoplasmic and nuclear proteins [15, 17]. However, the mechanism explaining how GZFLC modulates 14-3-3*γ*, FOXO, and TSC2 is poorly understood.

We undertook this study to observe the effects of GZFLC drug serum on proliferation and apoptosis of leiomyoma cells and to explore its mechanism. We hope to provide a new thread for effective treatment on uterine leiomyoma clinically.

## 2. Materials and Methods

### 2.1. Tissue Collection

Between October 2013 and July 2014, 20 patients who were diagnosed by B-ultrasound of uterine leiomyoma were recruited for the study. Patients' age ranged from 30 to 55 years. The types of surgery were uterine myomectomy and subtotal or total hysterectomy. No patients received any medication or hormonal therapy for at least 3 months prior to operation. Patients complicated with chronic diseases (such as hypertension and diabetes), infection, uterine malignancy, and adenomyosis (on the basis of tissue pathology) were excluded from the present study. The study protocol was approved by the Research Ethical Committee of the Second Affiliated Hospital of Wenzhou Medical University and written consent was obtained from patients before the collection of samples. The study met the standards of the Declaration of Helsinki.

### 2.2. Cell Primary Culture

Fresh tissues were thoroughly washed with phosphate-buffered saline (PBS) to remove blood. Then they were cut into small pieces (1 mm^3^) and placed into dissociation solution (Dulbecco's modified Eagle's medium (DMEM) and 0.2% v/v collagenase II (Invitrogen, Carlsbad, CA, USA)), followed by incubation for 4 h at 37°C in a water bath with continuous shaking to dissociate uterine leiomyoma cells. The dispersed cells were centrifuged at 100 ×g for 5 min. The resultant deposit was mixed with complete culture medium (DMEM, 10% fetal bovine serum, 100 IU/mL of penicillin G, and 100 *μ*g/mL streptomycin) and centrifuged at 100 ×g for 5 minutes. The resultant cells were plated at a density of 1 × 10^5^ cells per 60 mm dishes (Corning, USA) under 5% CO_2_ at 37°C in the complete culture medium. Culture medium was changed every other day. Cells were passaged as 1 : 2 every 4 to 6 days. Cells from third passage to the fifth one were used for all the experiments.

### 2.3. Preparation of Serum Containing the Tested Drugs

Subsequent to obtaining approval from the Ethics Committee of Wenzhou Medical University (Wenzhou, China), 60 male Sprague Dawley rats weighing 180–220 g were provided by the Experimental Animal Center of Wenzhou Medical University and housed in a room with a temperature of 21–25°C, relative humidity of 50–60%, and a 12-hour light/dark cycle. The recommended daily allowance of GZFLC tablet is 2.79 g for 60 kg body weight. The conversion ratio from a 70 kg man to a 200 g rat is 0.018 [18]; the corresponding dose of GZFLC tablet for rats was 0.293 g/kg per day. Therefore, rats were randomly divided into two groups, the experimental group was intragastrically given GZFLC (0.146 g/kg) twice daily (days 1–7) and the negative control group was intragastrically given same frequency with normal saline. Blood was acquired from the abdominal aorta of the rats 1 h after the last time of administration. The serum was collected by centrifugation (720 ×g for 20 min) and then filtered through a 0.22 *μ*m cellulose acetate membrane. Next, the serum was inactivated in 56°C water for 30 min and stored at −20°C until use.

In order to explain the method to make different concentrations of GZFLC serum, for example, 10 mL 20% GZFLC serum (complete culture medium containing 20% GZFLC serum) consisted of 7.9 mL DMEM, 2 mL rat serum with GZFLC treatment, 0.1 mL penicillin G, and streptomycin.

### 2.4. Cell Counting Kit-8 (CCK-8) Assay

The uterine leiomyoma cells were seeded in 96-well plates at 1 × 10^4^ cells/well and incubated in a 37°C, 5% CO_2_ incubator. After 24 h of seeding, the culture medium was removed and cells were accordingly treated. Subsequently, CCK-8 (Dojindo, Japan) at 10 *μ*L was added to each well and incubated for additional 2 h. The quantification was done by using spectrophotometry at a 450 nm wavelength, and the viability percentage was calculated as follows: (treated cells absorbent/nontreated cells absorbent) × 100.

### 2.5. Immunocytochemistry

Cells were washed with PBS and fixed in PBS containing 4% paraformaldehyde for 15 min, washed extensively with PBS after fixing and permeabilized in PBS containing 0.2% Triton X-100 for 15 min, and blocked in a serum-free blocking solution for 15 min at room temperature. Cells were then incubated with anti-*α*-smooth muscle actin antibody (1 : 100 dilution; Zhongshan Golden Bridge Biotechnology Co., Ltd., Beijing, China) overnight at 4°C. After PBS washing, cells were incubated with biotinylated rabbit anti-human IgG as secondary antibody. After incubation, the binding sites were visualised using 3,3′-diaminobenzidine. Finally, nuclei were stained with hematoxylin. Negative control slides, where primary antibody was replaced with PBS, were also included.

### 2.6. Annexin V-FITC/PI Assay

For apoptosis analysis, Annexin V-FITC/PI staining was performed using flow cytometry according to the manufacturer's guidelines. Briefly, cells were washed twice with cold PBS and then resuspended cells in ×1 binding buffer at concentration 1 × 10^6^ cells/mL. 10 *μ*L of the solution (1 × 10^5^ cells) was transferred to a 5 mL culture tube. 5 *μ*L of FITC Annexin V and 5 *μ*L PI were added. The cells were gently vortexed and incubated for 15 min at room temperature (25°C) in the dark. 40 *μ*L of ×1 binding buffer was added to each tube. Cells were analyzed by flow cytometry within 1 h.

### 2.7. Cell Lysate Preparation and Western Blot Analysis

Whole-cell lysates were prepared using ice-cold cell lysis buffer. Cells were collected and washed after indicated treatments. The protein from each experimental group was quantified by bicinchoninic acid (Beyotime, China). Cellular proteins (8 *μ*g) were solubilized in sample buffer (4% SDS, 30 mm dithiothreitol, 0.25 m sucrose, 0.01 m EDTA-Na_2_, and 0.075% bromophenol blue) and heated at 100°C for 5 min to denature proteins. The lysates were separated using electrophoresis on 12% sodium dodecyl sulfate-polyacrylamide gel and then electroblotted onto polyvinylidene fluoride membranes (Millipore, USA). The membranes were blocked for 2 h at room temperature in 0.05 M Tris-buffered saline with 0.5% triton X-100 (TBS-T, pH 7.4) containing 5% skimmed milk and then incubated with the appropriate primary antibody (anti-14-3-3*γ* (1 : 1000 dilution; Santa Cruz, USA), anti-FOXO (1 : 1000 dilution; CST, USA), anti-TSC2 (1 : 1000 dilution; CST, USA), and anti-*α*-Tubulin (1 : 2000 dilution; Beyotime, China)) in TBS-T overnight at 4°C. After washing with TBS-T, horseradish peroxidase-conjugated secondary antibodies (1 : 2000 for anti-rabbit IgG or anti-mouse IgG) for 1 h at room temperature, blots were developed by enhanced chemiluminescence. The expression levels of 14-3-3*γ*, FOXO, and TSC2 were quantified with densitometry and normalized by corresponding levels of *α*-Tubulin, respectively. Each experiment was repeated at least three times.

### 2.8. Statistical Methods

All statistical analyses were performed with SPSS17.0 software. If each group data was of normal distribution and homogeneity of variance, quantitative data was presented as the mean ± standard deviation; difference between two groups was analyzed by Student's *t*-test.

Difference among multiple groups was analyzed by one-way ANOVA, if variances were homogeneous; then least significance difference (LSD) method was used to compare between two groups. If variances were nonhomogeneous, Dunnett's T3 method was applied to compare between two groups.

Two-tailed *P* value < 0.05 was considered statistically significant.

## 3. Results

### 3.1. The Identification of Primary Culture Human Uterine Leiomyoma Cells

**Figure 1:**
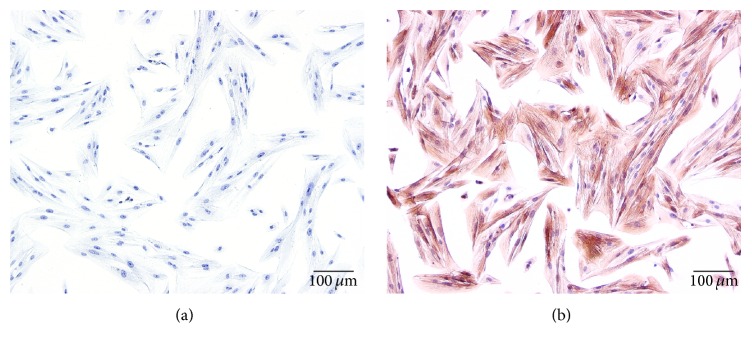
Staining of *α*-actin in human leiomyoma cells (SP staining, ×100). (a) Negative control; (b) *α*-actin positive staining.

To identify primary culture human uterine leiomyoma cells, immunocytochemistry staining of the cells with *α*-actin antibody was performed. As shown in Figure 1, positive brown signals of *α*-actin antibody mainly stained in cytoplasm, indicating that cell cultures derived from uterine leiomyoma tissues were identified correctly and retained their smooth muscle characteristics.

### 3.2. The Effect of GZFLC Drug Serum on Cell Proliferation of Human Uterine Leiomyoma Cells

As shown in Table 1, the negative control group compared with the blank control group (culture without serum); the negative control group at the saline serum concentration of 10%, 20%, and 30% and at time point of 24 h, 36 h, 48 h, and 72 h had higher optical density (OD) values (*P* < 0.05), indicating that rats serum promoted uterine leiomyoma cells proliferation. Compared with the negative control group, the GZFLC group showed that the OD value of 10% GZFLC serum at 36 h and 48 h was significantly lower (*P* < 0.05), the OD value of 20% GZFLC serum at 24 h, 36 h, and 48 h time point was obviously lower (*P* < 0.05), and at 72 h time point the inhibition phenomenon disappeared (*P* < 0.05); the OD value of 30% GZFLC serum at 12 h, 24 h, 36 h, and 48 h time point was significantly lower (*P* < 0.05) and at 72 h time point the inhibition phenomenon disappeared (*P* < 0.05), indicating that 30% GZFLC serum quickly inhibited cells proliferation and was effectively maintained for a long time. As the time point (12 h, 24 h, 36 h, and 48 h) increased, the OD value decreased with the drug concentration rise, but, at 72 h time point, the difference was not obvious, indicating that, within 48 hours, GZFLC serum inhibited human uterine leiomyoma cells proliferation was concentration-dependent and inhibitory effect weakened beyond that time.

### 3.3. The Effect of GZFLC Drug Serum on Cell Apoptosis of Human Uterine Leiomyoma Cells

**Figure 2:**
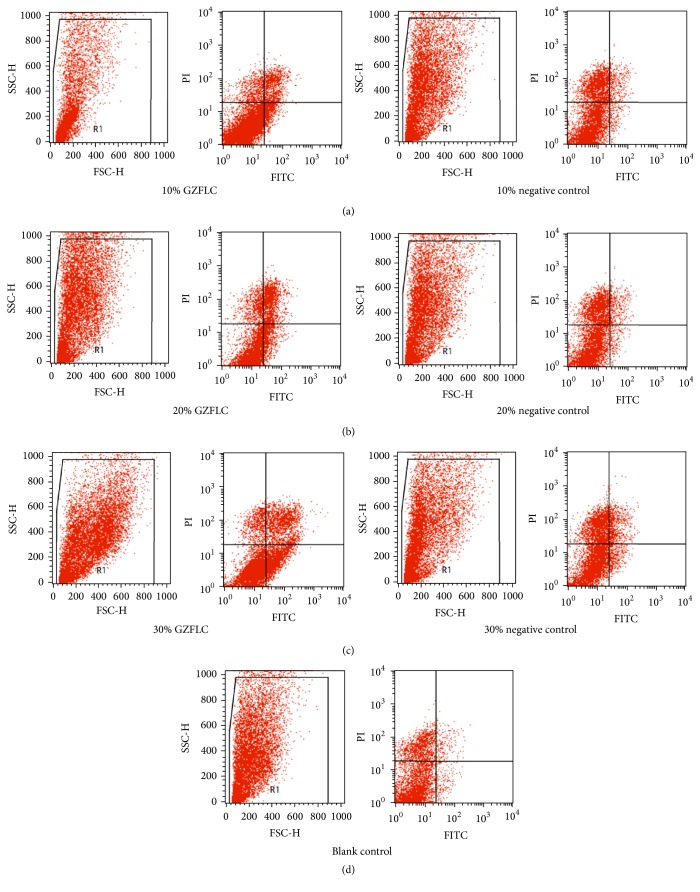
The cell apoptosis rate of different concentrations of GZFLC drug serum on the human leiomyoma cells via flow cytometry. (a) 10% GZFLC serum and 10% negative control. (b) 20% GZFLC serum and 20% negative control. (c) 30% GZFLC serum and 30% negative control. (d) Blank control.

**Figure 3:**
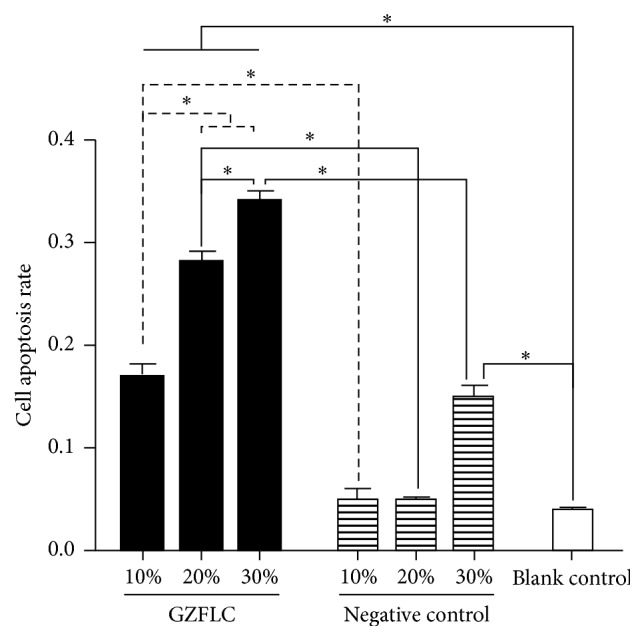
The cell apoptosis rate of different concentrations of GZFLC drug serum on the human leiomyoma cells. *∗* indicated *P* < 0.05.

As shown in Figures 2 and 3, the negative control group compared with the blank control group at the concentration of 10% and 20% (12 h) had no significant difference in cells apoptosis rate (*P* > 0.05), but at 30% it had increased apoptosis rate (*P* < 0.05), indicating that 30% rats blank serum promoted uterine leiomyoma cells apoptosis. Compared with the negative control group, the GZFLC group with different concentration (10%, 20%, and 30%) had higher cells apoptosis rate (*P* < 0.05). In addition, with the increased GZFLC serum concentration and cell apoptosis rate increasing, the different concentration within the apoptosis rate had significant difference (*P* < 0.05), indicating that GZFLC serum promoted human uterine leiomyoma cells apoptosis was concentration-dependent.

**Figure 4:**
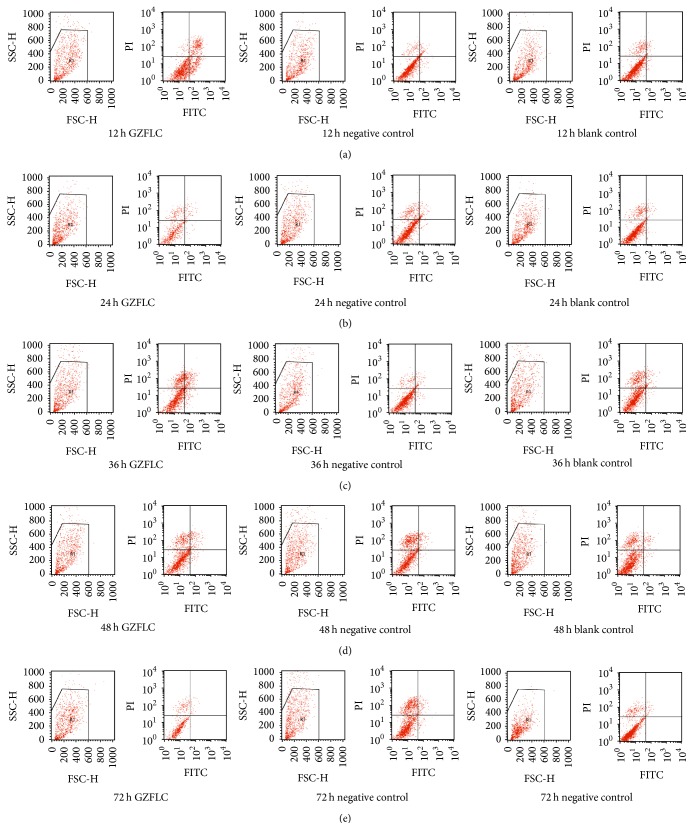
The cell apoptosis rate of GZFLC drug serum in different treatment points on the human leiomyoma cells via flow cytometry. (a) 12 h; (b) 24 h; (c) 36 h; (d) 48 h; and (e) 72 h.

**Figure 5:**
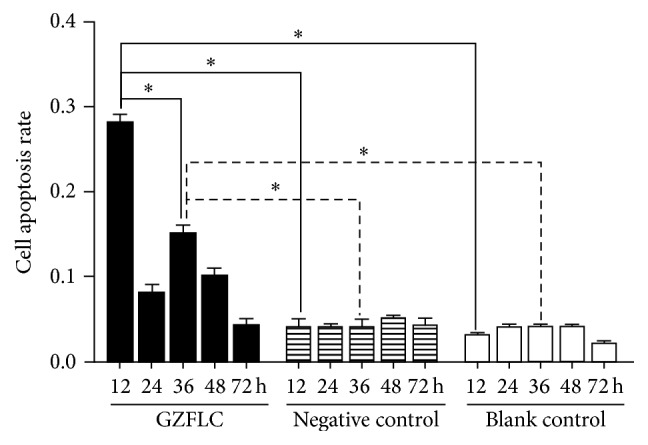
The cell apoptosis rate of GZFLC drug serum in different treatment points on the human leiomyoma cells (20% GZFLC serum concentration). *∗* indicated *P* < 0.05.

As shown in Figures 4 and 5, the negative control group compared with the blank control group at different time point (20% drug concentration) had no significant difference in cells apoptosis rate (*P* > 0.05), indicating that 20% rats blank serum had no obvious effects on cells apoptosis. Compared with the negative control group, the GZFLC group with 20% drug concentration had the most obvious apoptosis rate difference at 12 h, and then at 24 h it decreased, and after 36 h, apoptosis rate increased again (*P* < 0.05) and finally decreased.

GZFLC serum promoted human uterine leiomyoma cells apoptosis was concentration-dependent, 30% GZFLC serum had 34% apoptosis rate, but 30% rats blank serum also obviously promoted cells apoptosis (15% apoptosis rate); thus we chose 20% GZFLC serum as the optimal concentration to avoid the interference effect from rat blank serum. As the 20% drug concentration had the highest apoptosis rate at 12 h, it was considered as the optimal time point.

### 3.4. Effect of GZFLC Drug Serum on the Expression of TSC2, FOXO, and 14-3-3*γ* in the Human Leiomyoma Cells

**Figure 6:**
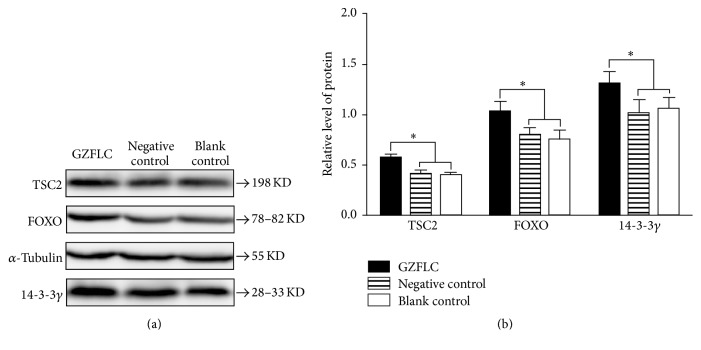
Effect of GZFLC drug serum on the expression of TSC2, FOXO, and 14-3-3*γ* in the human leiomyoma cells (12 h, 20% GZFLC serum concentration). (a) Western blot result. (b) Histogram result. *∗* indicated *P* < 0.05.

The proteins were collected at the optimal concentration and time point above. As shown in Figure 6, there was no significant difference in the expression of TSC2, FOXO, and 14-3-3*γ* proteins between the negative control group and the blank control group (*P* > 0.05). However, the GZFLC group had higher expression of TSC2, FOXO, and 14-3-3*γ* proteins compared with the negative control group (*P* < 0.05).

These results suggest that 14-3-3*γ* signal transduction pathway might be involved in GZFLC drug serum which inhibited the proliferation and induced apoptosis of uterine leiomyoma cells.

## 4. Discussion

In the present study, CCK-8 analysis and flow cytometry assay showed that GZFLC drug serum efficiently inhibited the proliferation and induced apoptosis of human uterine leiomyoma cells. In addition, Western blot revealed that this function may be related to the 14-3-3*γ*, TSC2, and FOXO pathway.

GZFLC is widely applied for uterine leiomyomas in China. Its traditional effects are invigorating blood, resolving masses, and dissolving stasis [8]. To assess the efficacy and safety of GZFLC for the treatment of uterine leiomyomas, Chen et al. identified 38 randomized controlled trials involving 3816 participants by meta-analyses [11] and found that GZFLC plus mifepristone was more effective than mifepristone alone in reducing the volume of uterine leiomyoma. GZFLC significantly improved symptoms of uterine leiomyoma, particularly in dysmenorrhea, when it was used alone or in combination with mifepristone. No serious adverse events were reported. However, the exact mechanism of GZFLC treatment for uterine leiomyomas is unclear.

Hu et al. reported that GZFLC attenuated endometriosis in rats via induction of apoptosis and inhibition of cell proliferation and metastasis [19]. In terms of its anticancer effect, this phenomenon was also found in human hepatocellular carcinoma [20, 21], bladder cancer [22], and cervical cancer [23]. As female rats have physiological cycle and the hormone levels and serum enzyme activity are relatively stable in male rats, male rats were selected in our experiments [24]. In this study, drug serum was chosen to simulate the drug effects on patients local tissue after medication; thus drug serum from rats after intragastric administration was applied to the primary culture cells, while normal saline serum was a negative control. In in vitro cell experiments with serum pharmacology, it is better to use the same species serum, as serums from different sources may interact with each other. In order to avoid the interference from fetal bovine serum, in this experiment, the rats serums were used to prepare different concentration with DMEM, which served as cell nutrients provider and drug carrier. GZFLC drug serum showed the inhibition of proliferation and induction of apoptosis on human uterine leiomyoma cells, which may explain that GZFLC could reduce the volume of leiomyomas and relieve the symptoms of leiomyomas. Compared to other time points, 12 h of GZFLC drug serum treatment was shown to be most effective in inducing cell apoptosis, indicating that the GZFLC serum has rapid efficacy. But the effects lasted a short time, at 24 h time point, the apoptosis rate decreased, and the possible explanation could be the increased amount of cell necrosis resulting in decreasing apoptosis rate. However, at 36 h, cell apoptosis rate rose again; the possibility that the second metabolic products of the compound traditional Chinese medicine worked could not be ruled out. Further research is needed.

In our previous study, 14-3-3*γ* was significantly downregulated in uterine leiomyoma compared to normal myometrium [16]; these results were then confirmed by several studies [25, 26], indicating that 14-3-3*γ* may play a role in the origin or growth of uterine leiomyomas. Wei et al. found that TSC2 was downregulated in uterine leiomyoma compared to normal myometrium from 60 hysterectomy specimens [27]. Kovács et al. reported that total FOXO1 protein exhibited nonsignificant difference, but phosphorylation FOXO1 protein was increased in leiomyoma compared with normal myometrium [25]. TSC2 and FOXO are regulated by specific interactions with 14-3-3 proteins [15]. In this study, we found that 14-3-3*γ* signal transduction pathway might be involved in GZFLC drug serum which inhibited the proliferation and induced apoptosis of uterine leiomyoma cells, but more researches are needed to confirm this link and elucidate the mechanisms.

## 5. Conclusions

The present study demonstrated that GZFLC drug serum inhibited the proliferation and induced apoptosis of uterine leiomyoma cells, which might be regulated by 14-3-3*γ* signal transduction pathway. These results may support further evaluation of a new thread for effective treatment on uterine leiomyoma clinically.

## Figures and Tables

**Table 1:** The effect of GZFLC drug serum on cell proliferation of human uterine leiomyoma cells.

Groups	Concentration (%)	Cases	OD value				
			12 h	24 h	36 h	48 h	72 h
GZFLC	10	6	0.60 ± 0.05	0.75 ± 0.04	0.86 ± 0.04^#^	1.06 ± 0.02^#^	1.27 ± 0.04^*∗*^
	20	6	0.60 ± 0.025	0.72 ± 0.02^#^	0.84 ± 0.02^#^	1.00 ± 0.06^#^	1.39 ± 0.022^#*∗*^
	30	6	0.58 ± 0.02^#^	0.69 ± 0.02^#^	0.78 ± 0.02^#^	0.91 ± 0.02^#^	1.49 ± 0.02^#*∗*^

Negative control	10	6	0.63 ± 0.05	0.80 ± 0.03^*∗*^	0.99 ± 0.05^*∗*^	1.19 ± 0.02^*∗*^	1.25 ± 0.03^*∗*^
	20	6	0.66 ± 0.03	0.89 ± 0.03^*∗*^	1.11 ± 0.05^*∗*^	1.28 ± 0.07^*∗*^	1.30 ± 0.02^*∗*^
	30	6	0.67 ± 0.03	0.92 ± 0.02^*∗*^	1.20 ± 0.01^*∗*^	1.32 ± 0.01^*∗*^	1.33 ± 0.01^*∗*^

Blank control	—	6	0.61 ± 0.03	0.70 ± 0.04	0.83 ± 0.05	0.90 ± 0.09	0.85 ± 0.08

^*∗*^Compared with blank control, *P* < 0.05.

^#^Compared with negative control, *P* < 0.05.
